# Insulin-like growth factor binding proteins in metabolic dysfunction-associated steatotic liver disease

**DOI:** 10.3389/fendo.2026.1853302

**Published:** 2026-06-05

**Authors:** Kuo Fang, Ji Miao, Guobin Song

**Affiliations:** 1 School of Medicine, The Chinese University of Hong Kong, Shenzhen, China; 2 Division of Endocrinology, Boston Children’s Hospital, Boston, MA, United States; 3 Department of Medicine, Harvard Medical School, Boston, MA, United States

**Keywords:** IGFBPs, MASLD, insulin resistance, lipid metabolism, inflammation, hepatic fibrosis, biomarkers

## Abstract

Metabolic dysfunction-associated steatotic liver disease (MASLD) is a highly prevalent condition that progresses from hepatic steatosis to steatohepatitis, fibrosis, and cirrhosis. While metabolic and inflammatory drivers of disease progression are well recognized, emerging evidence suggests that endocrine modulators, including the insulin-like growth factor binding proteins (IGFBPs), play important roles in MASLD. Beyond their canonical role as insulin-like growth factor (IGF) carriers, IGFBPs act as dynamic regulators of hepatic metabolism, inflammation, and fibrotic remodeling through both IGF-dependent and IGF-independent mechanisms. Growing evidence indicates that IGFBP1 and IGFBP2 confer metabolic protection by promoting lipid oxidation and increasing insulin sensitivity. IGFBP3 and IGFBP5 exhibit dual actions: they restrain lipogenesis at early stages but promote hepatocellular injury and stellate cell activation during fibrosis. IGFBP7 is a predominantly pathogenic modulator that impairs insulin signaling, drives ferroptosis, and fosters fibrosis. By contrast, IGFBP4 and IGFBP6 remain less well characterized. This review integrates recent mechanistic and translational findings on IGFBPs in MASLD with evidence accumulated from recent studies, highlighting their potential as biomarkers for disease staging and as therapeutic targets for interventions.

## 1 Introduction

Metabolic dysfunction-associated steatotic liver disease (MASLD), formerly known as nonalcoholic fatty liver disease (NAFLD), is a spectrum of liver pathology driven by metabolic syndrome-related factors such as insulin resistance, obesity, dyslipidemia, and chronic low-grade inflammation (1, 2). It is a progressive disorder comprising a broad hepatic pathology ranging from simple steatosis, steatohepatitis, fibrosis, cirrhosis, and hepatocellular carcinoma (HCC) (3, 4). The prevalence of MASLD is rising in parallel with global trends in obesity and type 2 diabetes (T2DM); therefore, it is now considered to be a major public health concern with limited therapeutic options (5, 6). At the molecular level, the pathogenesis of MASLD involves complex interactions among dysregulated lipid metabolism, insulin resistance, inflammatory cascades, hepatocyte injury, immune cell activation, dysbiosis of the gut microbiota, and fibrogenic transformation (7, 8). Recent studies have increasingly implicated canonical insulin-like growth factor binding proteins (IGFBPs), a family comprising six structurally conserved proteins (IGFBP1 to IGFBP6), as well as IGFBP-related protein 1 (IGFBP7), in the disease.

Structurally, IGFBPs contain conserved N-terminal and C-terminal domains connected by a variable linker, which collectively form IGF-binding pockets that regulate bioavailability and tissue distribution of IGF (9–11). The N-terminus of canonical IGFBPs shares an evolutionarily conserved cysteine-rich domain that forms a specific disulfide-bonded structure essential for interactions with insulin-like growth factor I (IGFI) and insulin-like growth factor II (IGFII) (11–14). By contrast, the N-terminal sequence of IGFBP7 has fewer cysteines with altered arrangements, leading to a lower affinity for IGF than that of canonical IGFBPs (11, 12). The C terminus of the IGFBPs acts as a functional module that maintains a conserved fold, which is crucial for ligand interactions and protein stability despite limited sequence conservation compared with the N-terminal domain (13). For instance, IGFBP2 contains an Arg-Gly-Asp (RGD) integrin-binding motif, enabling direct interaction with integrins such as α5β1 and thereby modulating cell adhesion and migration (15–17). IGFBP3 and IGFBP5 contain nuclear localization signals (NLS) within the C-terminal domain, allowing nuclear translocation and transcriptional regulation (13, 18–20). In addition, the heparin-binding domains (HBDs) present in IGFBP2, IGFBP5, and IGFBP7 mediate binding to glycosaminoglycans and ECM components, supporting local growth factor reservoir functions (21, 22). The linker region is the most variable region among IGFBPs, enabling post-translational modifications. For instance, phosphorylation of IGFBP3 within the linker region reduces its binding to IGFI while enhancing IGF-independent activity (23). Collectively, these structural features enable IGFBPs to regulate the bioavailability and distribution of IGF, while at the same time exerting IGF-independent actions that overlap with hallmarks of MASLD progression, such as lipid metabolism, insulin sensitivity, oxidative stress, inflammation, and extracellular matrix (ECM) remodeling (24, 25). The domain architecture of the IGFBP family is summarized in **Table 1**.

**Table 1:** Structural domains and characteristic motifs of IGFBP1–7.

IGFBP	N-terminal	Linker (central)	C-terminal	References
IGFBP1	Cysteine-rich domain forming disulfide bonds	Variable flexible region	RGD motif	(14, 26)
IGFBP2	Cysteine-rich domain	Variable linker region	RGD motif HBD domain	(11, 14, 15, 27)
IGFBP3	Cysteine-rich domain	Contains phosphorylation sites	NLS domain HBD domain ALS-binding site	(14, 18, 28)
IGFBP4	Cysteine-rich domain	Variable region with proteolytic cleavage site (PAPP-A sensitive)	—	(14, 29–31)
IGFBP5	Cysteine-rich domain	Variable linker region	NLS domain HBD domain	(14, 18, 21)
IGFBP6	Cysteine-rich domain with strong IGF-II affinity	Variable linker region	—	(11, 14, 32)
IGFBP7	Reduced cysteine number; Altered cysteine pattern	Variable linker region	C-terminal organization distinct from that of canonical IGFBPs	(11, 12, 14)

This table summarizes the domain architecture and conserved motifs of the IGFBP family. All canonical IGFBPs (IGFBP1–6) share a modular organization, with a conserved N-terminal cysteine-rich IGF-binding domain, a variable central linker region that contains protease-sensitive cleavage sites, and a C-terminal domain contributing to high-affinity IGF binding and interaction with extracellular matrix components.

Distinct motifs: the RGD (integrin-binding) sequence in IGFBP1 and IGFBP2; the heparin-binding domains (HBDs) in IGFBP2, IGFBP3, and IGFBP5; the acid-labile subunit (ALS)-binding sites in IGFBP3; the pregnancy-associated plasma protein-A (PAPP-A) sensitive region in IGFBP4; and the nuclear localization signal (NLS) in IGFBP3 and IGFBP5. IGFBP7 (IGFBP-related protein) diverges structurally, with a reduced number of cysteines and distinct C terminal organization, leading to relatively low affinity for IGF and distinct extracellular regulatory functions.

Notably, accumulating evidence indicates that expression of individual IGFBPs is dynamically regulated across different stages of MASLD, reflecting their distinct functional roles during disease progression. These stage-specific expression patterns are summarized in **Table 2**. Despite growing evidence, their roles in MASLD have not been systematically summarized and categorized across the literature. Thus, we provide here a timely and comprehensive summary of how IGFBP family members contribute to the initiation, progression, and potential resolution of MASLD. This review synthesizes recent mechanistic and translational evidence highlighting the dual protective and pathogenic roles of IGFBPs in disease progression, as well as their potential as biomarkers and therapeutic targets.

**Table 2:** Stage-specific expression patterns of IGFBP1–7 during MASLD progression.

IGFBP	Steatosis	MASH	Fibrosis	Reference
IGFBP1	Human hepatocytes ↑ Human serum ↓ MCD-diet mouse liver ↑	—	Human plasma ↑	(29, 33–42)
IGFBP2	Human liver/serum ↓ HFD-diet mouse liver ↓	Human NASH liver ↓	Human serum ↓	(43–49)
IGFBP3	Human hepatocytes/serum ↓	—	Human serum ↓ HSC of mouse models (CCl_4_/BDL) ↑	(9, 28, 50–57)
IGFBP5	Human hepatocytes ↓ HFD-diet mouse liver ↓	Human serum **↑**	Human serum **↑** HSC of mouse models (CCl_4_/BDL) **↑**	(58–63)
IGFBP7	Human liver ↑ HFD-diet mouse liver ↑ zebrafish MASLD model ↑	Human serum/liver **↑**	Human serum (F3–F4) **↑** HSC of mouse **↑**	(13, 29, 64–73)
IGFBP4	—	—	—	
IGFBP6	Human liver ↑	—	HSC of mouse model **↑**	(64, 74, 75)

This table summarizes dynamic changes in IGFBP expression across major histological stages of MASLD, including steatosis, steatohepatitis, and fibrosis. Upward arrow ↑ indicates an increase or upregulation, whereas downward arrow ↓ indicates a decrease or downregulation of IGFBP expression.

## 2 Progression and pathogenesis of MASLD

MASLD is an overarching term that has replaced NAFLD because the disease is highly pertinent to metabolic dysregulation (76). Currently, it affects about 38% of adults worldwide, with prevalence projected to reach 55% by 2040 due to rising rates of obesity, insulin resistance, and sedentary lifestyles (6). Recent data from the Global Burden of Disease study estimates that over 1.27 billion individuals globally live with MASLD in 2021, with approximately 48.35 million new cases annually, highlighting its rapid growth and increasing healthcare burden (77). Notably, a recent study indicates that a significant proportion of MASLD cases remain undiagnosed, with approximately 79.8% of patients in the United Kingdom lacking diagnosis (78). In a Canadian study comparing tertiary-care diagnosed patients with community-screened undiagnosed patients, the undiagnosed group was older, more often female, less frequently diabetic, more frequently dyslipidemic, and had milder biochemical and non-invasive markers of liver disease severity (79). These findings highlight the urgent need for reliable biomarkers to improve early detection and risk stratification.

Both metabolic and environmental factors promote MASLD. Obesity, T2DM, and insulin resistance are strongly associated with MASLD. Obesity and a higher body mass index (BMI) are strongly associated with MASLD in a dose-dependent manner, with the risk increasing by approximately 20% for each 1-unit increase in BMI (80). T2DM also increases MASLD risk and severity significantly, with approximately 70% of patients with T2DM developing MASLD (81, 82). Obesity also promotes insulin resistance, which disrupts glucose and lipid metabolism, promotes hepatic fat accumulation, and induces adipose tissue dysfunction with increased pro-inflammatory cytokines, thereby accelerating liver damage (81). Dietary factors such as high intake of saturated fats and fructose also contribute to hepatic lipid accumulation by promoting *de novo* lipogenesis and mitochondrial dysfunction (83).

MASLD begins with hepatic steatosis, progresses to inflammation and hepatocellular injury (both of which characterize metabolic dysfunction-associated steatohepatitis (MASH)), and ultimately advances through fibrogenesis to cirrhosis or HCC (84). During the initial stage, excessive lipid accumulation within hepatocytes arises due to increased uptake of free fatty acid (FFA), enhanced *de novo* lipogenesis, and impaired secretion of very-low-density lipoprotein (VLDL) (83). This lipid overload induces lipotoxicity, characterized by the accumulation of toxic lipid species, particularly saturated fatty acids such as palmitic acid, which disrupt cellular homeostasis, trigger hepatocellular stress responses, and ultimately induce steatohepatitis (85, 86). As the disease progresses to MASH, lipotoxicity induces endoplasmic reticulum (ER) stress, thereby activating unfolded protein response pathways and upregulating pro-apoptotic factors (87, 88). Meanwhile, mitochondrial dysfunction impairs fatty acid β-oxidation and promotes excessive production of reactive oxygen species (ROS), leading to oxidative stress and further hepatocyte injury (83). Persistent hepatocellular stress activates Kupffer cells and hepatic stellate cells (HSCs), leading to the release of pro-inflammatory cytokines, including tumor necrosis factor-alpha (TNF-α) and interleukin 6 (IL-6), as well as profibrotic mediators such as transforming growth factor-beta (TGF-β), which together drive lobular inflammation and initiate fibrogenic responses (89). Multiple forms of regulated cell death, including apoptosis, necrosis, and ferroptosis, contribute to progressive loss of hepatocytes, with ferroptosis becoming increasingly prominent in the advanced stages (83). During the late stage of MASLD, disease progression is marked by persistent chronic inflammation, fibrotic remodeling, and worsening metabolic dysfunction. Activation of HSCs becomes a key event, leading to their transdifferentiation into myofibroblasts and subsequent excessive ECM deposition, which defines clinically significant fibrosis and predicts adverse outcomes (90–92). This fibrogenic response correlates strongly with the severity of insulin resistance, forming a vicious cycle that propels the transition to cirrhosis and HCC (93).

Progression of MASLD is further shaped by extrahepatic conditions that exacerbate liver injury and metabolic dysfunction. A notable extrahepatic comorbidity that amplifies disease severity is obstructive sleep apnea (OSA), which induces chronic intermittent hypoxia. This hypoxic stress amplifies systemic inflammation and aggravates hepatocellular injury, thereby accelerating disease progression (94). Concurrently, dysbiosis of the gut microbiota, frequently observed in cases of advanced MASLD, further exacerbates hepatic injury by increasing intestinal permeability, thereby allowing bacterial products such as lipopolysaccharide (LPS) to translocate to the portal circulation (89). These endotoxins activate hepatic immune responses and amplify inflammation, further driving the transition to cirrhosis and, in some cases, HCC (95, 96). Sarcopenia, another prevalent comorbidity observed during late-stage MASLD, imposes additional metabolic burden by diminishing skeletal muscle mass, which impairs glucose homeostasis and intensifies insulin resistance, thereby reinforcing disease progression (97).

**Figure 1:**
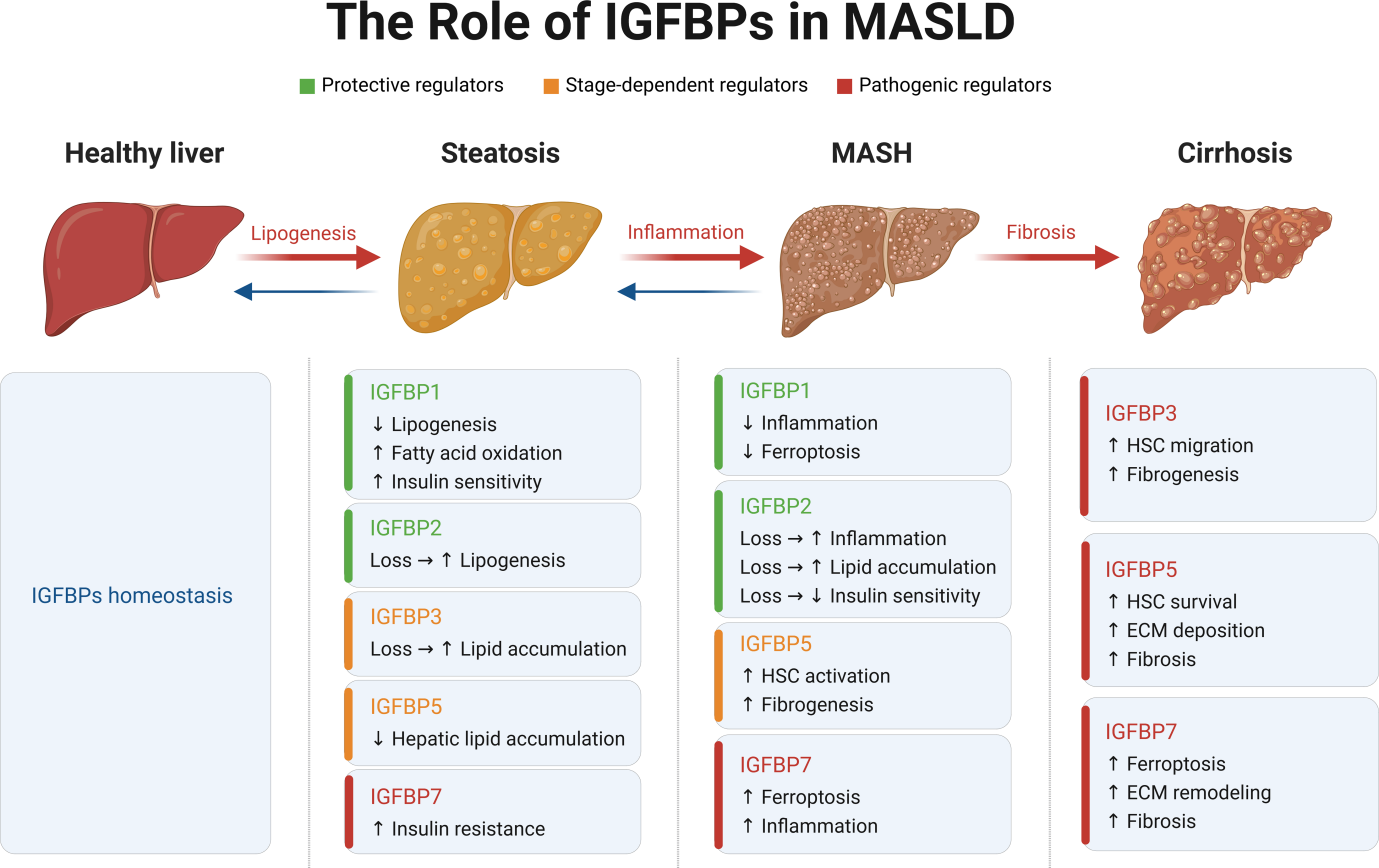
The role of IGFBPs in MASLD. Schematic overview of the functions of IGFBPs across MASLD progression. IGFBP1 and IGFBP2 primarily regulate lipid metabolism and insulin sensitivity during steatosis and MASH. IGFBP3 and IGFBP5 exhibit stage-dependent roles, contributing to lipid regulation in early disease and to fibrogenic responses in later stages. IGFBP7 is associated with insulin resistance, inflammation, ferroptosis, and fibrosis. Loss indicates effects associated with reduced IGFBP expression or function. Colors denote protective, stage-dependent, and pathogenic regulators. Arrows indicate increased or decreased biological processes. HSC, hepatic stellate cell; ECM, extracellular matrix; MASH, metabolic dysfunction-associated steatohepatitis. Upward arrow ↑ indicates an increase or upregulation, whereas downward arrow ↓ indicates a decrease or downregulation in pathway activity or biological processes shown in the figure. Created in BioRender. Fang, K. (2026) https://BioRender.com/d6pkzyf. License: BioRender Publication and Licensing Rights – Open Access; Agreement number: CE29ORIB22. Source: BioRender.com. The figure was created using BioRender and was not reproduced or adapted from a previously published figure.

IGFBPs regulate glucose metabolism, lipid homeostasis, inflammation, and fibrosis—key processes central to MASLD pathogenesis. Accordingly, IGFBP dysregulation in MASLD is closely associated with disease initiation, progression, and metabolic remodeling. Although individual IGFBP family members may exert distinct effects across disease stages and cellular contexts, their altered expression supports their potential relevance as both biomarkers and therapeutic targets in MASLD (**Figure 1**).

## 3 Protective modulators: IGFBP1 and IGFBP2

Within the IGFBP family, IGFBP1 and IGFBP2 are closely tied to insulin signaling and nutrient availability, and their expression levels fluctuate in response to metabolic status. During the early stage of MASLD, which is driven by insulin resistance and lipid overload, IGFBP1 and IGFBP2 appear to exert predominantly protective roles by modulating hepatic fat accumulation and improving insulin sensitivity.

### 3.1 IGFBP1

IGFBP1, a 30 kDa protein synthesized primarily by hepatocytes and secreted into the circulation (98, 99), binds to IGFs to regulate their bioavailability and exhibits increased binding affinity for IGFI when phosphorylated (100). IGFBP1 affects cellular processes independently of the IGF receptor pathway by interacting with integrin α5β1 through its C-terminal RGD motif (26).

In healthy individuals, circulating IGFBP1 levels respond acutely to changes in insulin concentration (33), falling postprandially due to insulin suppression, and rising during fasting (34, 101). By contrast, in MASLD patients, IGFBP1 exhibits stage-specific expression dynamics. Some studies show that during the early and intermediate stages of MASLD, expression of both mRNA and protein levels of IGFBP1 is elevated. FFA-exposed human hepatocytes show sustained elevation of IGFBP1, which suppresses nuclear factor kappa-light-chain-enhancer of activated B cells (NF-κB)-driven inflammation (35). Elevated hepatic *Igfbp1* expression is also observed in methionine- and choline-deficient (MCD) diet-fed mice, in which it correlates positively with liver fat content and inhibits lipogenesis (35). While hepatic IGFBP1 protein levels rise locally, circulating IGFBP1 levels may decrease due to systemic insulin resistance, as evidenced by human studies (36, 64, 102); however, another study showed that hepatic *Igfbp1* gene expression is downregulated in high-fructose and high-fat diet (HFD)-fed mice (103). During the late stage of MASLD, plasma IGFBP1 is elevated, and its levels correlate with advanced fibrosis in human MASLD (37, 104).

During the early and intermediate stages of MASLD, upregulation of IGFBP1 exerts hepatoprotective effects, whereas deficiency exacerbates disease progression. Studies show that IGFBP1 plays protective roles by improving lipid metabolism, suppressing inflammation, and improving insulin sensitivity. Recent studies show that increasing IGFBP1 during the early stage of MASLD ameliorates steatosis by modulating lipid metabolic pathways (35). In C57BL/6 mice fed a MCD diet, administration of recombinant (r)IGFBP1 reduces hepatic steatosis significantly by downregulating the mRNA and protein expression of sterol regulatory element-binding protein 1 (SREBP1), as well as its target genes, including acetyl-CoA carboxylase (*Acc*) and fatty acid synthase (*Fasn*) (35). Administration of rIGFBP1 also enhances fatty acid β-oxidation through increased levels of peroxisome proliferator-activated receptor α (PPARα) protein. This nuclear transcription factor activates genes involved in mitochondrial and peroxisomal fatty acid β-oxidation, such as carnitine palmitoyltransferase 1α (*Cpt1α*), which encodes the rate-limiting mitochondrial enzyme responsible for transporting long-chain fatty acids into mitochondria. In parallel, PPAR gamma coactivator-1α (PGC1α), which potentiates PPARα transcriptional ability, is also upregulated. Consistent with these results, IGFBP1 knockdown using small interfering RNA (siRNA) downregulates proteins involved in fatty acid β-oxidation and upregulates proteins involved in lipid synthesis (35). During the intermediate stage of MASLD, characterized by hepatic inflammation, IGFBP1 suppresses pro-inflammatory signaling by inhibiting NF-κB and extracellular signal-regulated kinase (ERK) signaling by binding to integrin β1 via its RGD motif, thereby alleviating hepatocellular injury (35). Conversely, IGFBP1 deficiency worsens MASLD significantly, as evidenced by studies demonstrating that loss of IGFBP1’s hepatoprotective effects leads to accelerated lipid deposition and elevation of inflammatory cytokines (29, 35).

Additionally, IGFBP1 improves insulin sensitivity and prevents ferroptotic cell death, while simultaneously mitigating hepatic lipid accumulation (38, 39). In a mouse model fed with MCD and a HFD, IGFBP1 upregulation caused by *Serpina3n* deficiency, which was accompanied by increased leptin receptor (*Lepr*) mRNA expression, and activation of LEPR-signal transducer and activator of transcription 3 (STAT3) pathway, attenuates hepatic steatosis, and improves insulin sensitivity (39). Similarly, in diabetic mice fed a Western diet, administration of Salvianolic acid A (SAA) restores hepatic IGFBP1 expression through activation of the AMP-activated protein kinase (AMPK) pathway (38). This activation counteracts insulin-mediated suppression of IGFBP1 and improves metabolic homeostasis by modulating lipid synthesis and oxidation, thereby suppressing ferroptosis and reducing hepatic lipid accumulation. Importantly, knockdown of IGFBP1 using siRNA completely abolishes SAA-mediated inhibition of lipid peroxidation, steatosis, and ferroptosis in mice, despite persistent AMPK activation, indicating that IGFBP1 mediates the effects of AMPK on steatosis, inflammation, and ferroptosis (38). It is also worth noting that lifestyle intervention-induced changes in IGFBP1 through hypocaloric or high-fiber diets, as well as structured lifestyle programs, are associated with reduced intrahepatic triglyceride content, reflecting beneficial metabolic adaptations (34). Consistently, IGFBP1 promotes lipid oxidation and improves mitochondrial efficiency in hepatocytes under nutrient-deprived conditions (40). However, excessive or sustained overexpression of IGFBP1 may contribute to pathogenic effects through the IGF axis. In transgenic mice with ubiquitous, constitutive overexpression of IGFBP1 driven by the phosphoglycerate kinase promoter, chronic supraphysiological IGFBP1 attenuates the hypoglycemic actions of IGFI, disrupts glucose homeostasis, and potentially induces insulin resistance (41). These findings suggest that IGFBP1 may have a dose-dependent effect, in which moderate physiological increases are protective, but chronic dysregulation may contribute to metabolic imbalance.

Studies suggest that IGFBP1, particularly fasting or phosphorylated IGFBP1, has potential as a predictive biomarker for MASLD. Dynamic changes in circulating IGFBP1 following metabolic challenges closely reflect hepatic insulin sensitivity, providing an early and sensitive indicator of metabolic dysfunction (42). In pediatric populations, lower IGFBP1 levels are associated with increased obesity, insulin resistance, and dietary risk factors such as high fructose intake, underscoring its relevance across age groups (105–107). In addition, measuring phosphorylated IGFBP1 improves the accuracy of non-invasive models for predicting liver fat content, outperforming conventional indices based solely on liver enzymes (108). Collectively, these findings suggest that IGFBP1, through its dynamic regulation, phosphorylation status, and close association with metabolic and dietary factors, may be used as a versatile biomarker for assessing hepatic insulin resistance and predicting MASLD progression.

### 3.2 IGFBP2

IGFBP2, a 31.4 kDa protein, is the second most abundant IGFBP in the circulation, and is expressed abundantly in metabolically active tissues such as liver, adipose tissue, and pancreas (13, 109, 110). Both clinical and preclinical studies consistently demonstrate significant downregulation of IGFBP2 expression during the early and intermediate stages of MASLD. In liver samples from humans with MASLD presenting as simple steatosis, the mRNA and protein expression of IGFBP2 is reduced markedly (43). During the intermediate stage of MASLD, *IGFBP2* is one of the most significantly downregulated hepatic genes, with further reductions in MASH patients relative to steatotic patients (43, 44). Reduced circulating IGFBP2 levels are strongly associated with increased hepatic fat content, higher BMI, insulin resistance, and visceral adiposity (45, 46, 111). In individuals with obesity, lower IGFBP2 levels predict MASLD incidence, and genetic evidence supports a causal relationship between IGFBP2-related pathways and MASLD risk (45, 47, 111). Decreased expression of the *IGFBP2* gene in MASLD is likely linked to promoter hypermethylation (epigenetic silencing) and post-transcriptional repression by microRNAs such as miR-130b-5p, which directly targets *Igfbp2* mRNA in murine MASLD models (44, 48, 112). Notably, hypermethylation of the *IGFBP2* gene promoter is observed consistently in human MASLD, particularly in adolescent cohorts with early-stage steatosis, as well as that of *Igfbp2* in HFD-fed mouse models (46, 48). This hypermethylation, primarily mediated by recruitment of DNA methyltransferase 3 alpha (DNMT3A) to the *Igfbp2* promoter, results in persistent transcriptional silencing of *Igfbp2* (48).

IGFBP2 acts as a crucial endogenous hepatoprotective regulator by binding directly to the epidermal growth factor receptor (EGFR), thereby suppressing downstream STAT3 phosphorylation (43). Inhibition of the EGFR-STAT3 signaling cascade prevents transcription of sterol regulatory element-binding transcription factor which encodes SREBP1, thereby reducing transcription of lipogenic genes such as *Fasn* and *Scd1*, and mitigating hepatic lipid accumulation in HFD-fed mice (43). Notably, STAT3 has been reported to exert divergent effects in liver disease. Depending on the upstream activating signal, cell type, duration of signaling, metabolic state, and downstream transcriptional program, STAT3 activation can be either hepatoprotective or steatogenic (113). Thus, the protective role of STAT3 in the EGFR–STAT3 pathway is distinct from its lipogenic role in the *Serpina3n*-deficient model, in which IGFBP1 levels were upregulated, highlighting the complex, context-dependent effects of STAT3 signaling in MASLD. Furthermore, during the early stage of MASLD in HFD-fed mice, suppression of IGFBP2 expression by DNMT3A-mediated promoter hypermethylation initiates a pathological cascade in which inadequate inhibition of SREBP1 maturation promotes uncontrolled hepatic lipogenesis (48). By contrast, restoration of IGFBP2 by fibroblast growth factor 1 (FGF1)-induced demethylation improves insulin sensitivity markedly and attenuates lipogenic gene transcription. As MASLD progresses toward MASH, persistent *Igfbp2* deficiency significantly exacerbates hepatic lipid accumulation in HFD-fed mice (48). Furthermore, during the intermediate stage of MASLD, gut dysbiosis drives a further decline in IGFBP2 levels, particularly through overgrowth of *Enterococcus faecalis*, which downregulates the FGF1-IGFBP2 signaling axis and disrupts downstream insulin receptor substrate 1 (IRS1)/CPT1α-mediated metabolic pathways, resulting in impaired insulin sensitivity and persistent hepatic inflammation (114).

Preclinical studies indicate that interventions capable of restoring IGFBP2 expression may hold therapeutic potential for MASLD. Pharmacological treatments such as recombinant FGF1 (rFGF1) reverse *Igfbp2* promoter hypermethylation by inhibiting DNMT3A recruitment, thereby elevating *Igfbp2* expression and ameliorating lipid accumulation in the liver of HFD-fed mice (48). Similarly, protocatechuic acid supplementation improves MASLD in murine models by modulating the gut-liver axis. It does this by reducing *Enterococcus faecalis* abundance and upregulating hepatic *Igfbp2* expression, accompanied by restoration of IRS1-associated insulin signaling and CPT1α-mediated fatty acid oxidation (114). Collectively, these findings support IGFBP2 as a metabolic regulator whose depletion accelerates disease progression, whereas restoring its expression reverses steatosis and improves systemic metabolic health.

Additionally, IGFBP2 may be a promising biomarker for MASLD, particularly in asymptomatic individuals. Recent studies demonstrate that when combined with anthropometric indices such as BMI and waist circumference, circulating IGFBP2 levels predict MASLD in the early stage, with a sensitivity of 73.5% and a specificity of 75.2%, particularly in asymptomatic individuals (49). Additional research is required to clarify its standalone diagnostic utility, and to compare its performance with that of established markers such as alanine aminotransferase (ALT), aspartate aminotransferase (AST), or imaging-based modalities. Future studies should evaluate these comparisons to better define the diagnostic advantages of IGFBP2.

In conclusion, IGFBP1 and IGFBP2 are protective modulators for MASLD. IGFBP1 responds acutely to insulin fluctuations and plays a protective role by regulating lipid metabolism, improving insulin sensitivity, and suppressing inflammation, particularly during the early and intermediate disease stages; however, excessive or sustained overexpression of IGFBP1 may disrupt glucose homeostasis and exacerbate steatosis. IGFBP2 is consistently downregulated across MASLD cohorts, where its loss promotes hepatic lipogenesis, insulin resistance, and inflammation through activation of the EGFR-STAT3 pathway and suppression of the gut microbiota-driven FGF1-IGFBP2 axis. Restoring IGFBP2 levels ameliorates steatosis and improves systemic insulin sensitivity, lipid oxidation, and overall metabolic homeostasis. Furthermore, IGFBP1 reflects hepatic insulin sensitivity and may enhance the utility of diagnostic models, whereas circulating IGFBP2 serves as a promising biomarker, particularly when integrated with anthropometric indices such as waist circumference. Taken together, studies to date suggest that IGFBP1 and IGFBP2 actively modulate key mechanisms underlying MASLD pathogenesis, and as such represent promising targets for early diagnosis and therapeutic intervention.

## 4 Stage-dependent modulators: IGFBP3 and IGFBP5

While IGFBP1 and IGFBP2 act during the early and intermediate stages of MASLD, IGFBP3 and IGFBP5 are strongly associated with the late stages of disease progression, which involve hepatocellular injury, apoptosis, and fibrogenesis.

### 4.1 IGFBP3

IGFBP3 is a 28 kDa glycoprotein that contains a C-terminal domain featuring heparin-binding motifs, as well as the acid-labile subunit (ALS) interaction interface (28, 115, 116) that enables IGFBP3 to form ternary complexes with IGFs and ALS in the circulation, thereby prolonging the half-life of IGF and restricting its bioavailability to target tissues (9, 50, 117). Proteolytic fragments of the N-terminal 1–95 amino acid region demonstrate potent growth-inhibitory effects, while the intact protein modulates cellular glucose uptake via its C-terminal HBD (28, 115). Furthermore, the C-terminus contains a functional NLS that permits nuclear translocation and directs transcriptional coregulation, which includes the p53-dependent pro-apoptotic programs and SMAD-mediated signaling; in addition, the C-terminus of IGFBP3 can activate a caspase-8-mediated apoptosis pathway via the transmembrane protein 219 receptor. In hepatocytes, IGFBP3 further dampens stress-kinase and inflammatory signaling under lipotoxic conditions (51, 118), in which overexpression of IGFBP3 alleviates lipotoxic inflammation by suppressing the c-Jun N-terminal kinase (JNK) and NF-κB signaling pathways.

In humans, IGFBP3 is produced predominantly by the liver (under growth-hormone (GH) control) and is the principal serum carrier for IGFI and IGFII, thereby buffering IGF bioavailability systemically (14, 119). IGFBP3 expression declines progressively from early steatosis to the advanced stages of MASLD. In oleic acid-treated human hepatocytes that mimic hepatic steatosis, hepatocytes show marked downregulation of IGFBP3 at both the mRNA and protein levels, alongside increased levels of intracellular triglycerides, a finding consistent with disruption of the GH-IGF axis (52). As MASLD progresses to steatohepatitis and fibrosis, clinical studies reveal a further decline in systemic IGFBP3 bioavailability, as evidenced by reduced serum concentrations of IGFBP3 (53, 54). This continuum of suppression mechanistically links IGFBP3 dysregulation to MASLD pathogenesis, in which progressive loss of its regulatory function exacerbates lipid accumulation.

IGFBP3 contributes to MASLD progression in a complex manner, appearing to act as a stage-dependent modulator during pathogenesis. Human genetic studies identify the *IGFBP3* promoter variant rs2854744 as a risk factor for MASLD; indeed, it confers an approximately 2.7-fold increase in susceptibility. The risk allele reduces both promoter activity and circulating IGFBP3 levels, thereby establishing a direct genetic connection to dysregulated IGF-axis signaling (55, 120). During early steatosis, global *Igfbp3* knockout mice fed a HFD exhibit increased fasting glucose and insulin levels, elevated basal hepatic glucose production in hyperinsulinemic clamp studies, and pronounced hepatic steatosis (56). These observations suggest that loss of IGFBP3 disrupts both glucose homeostasis and lipid regulation in the liver, reinforcing its hepatoprotective role; however, bile duct ligation (BDL)-induced fibrosis in *Igfbp3*-deficient mice leads to significantly lower collagen deposition than in wild-type (WT) controls, suggesting a pro-fibrogenic role for IGFBP3 in hepatic fibrogenesis and vascular remodeling (57). Mechanistically, IGFBP3 drives HSC migration through a β1-integrin-AKT pathway that is enhanced by iron and is largely IGF-independent, thereby enhancing fibrogenic tone (57). Although these findings were obtained after cholestatic injury rather than from MASLD models, they indicate that IGFBP3 may promote fibrogenic processes as metabolic liver disease progresses toward advanced fibrotic stages. Collectively, these data suggest that IGFBP3 may limit lipid accumulation during early metabolic steatosis, yet promotes HSC-driven fibrogenesis and injury as the disease progresses.

Additionally, in adults with biopsy-proven MASLD, a previous study reported that circulating IGFI and the IGFI/IGFBP3 ratio associate independently with fibrosis severity, supporting their use as non-invasive biomarkers of advanced histology (53). These findings were corroborated in pediatric cohorts in which decreased serum IGFBP3 and a disordered IGFI/IGFBP3 balance aligned with greater disease severity (54).

### 4.2 IGFBP5

IGFBP5 is a secreted glycoprotein consisting of 272 amino acids, with a molecular weight of approximately 30.6 kDa. At the early stage of steatosis in MASLD, IGFBP5 expression is consistently downregulated, as shown in preclinical models, including FFA-treated HepG2 cells and HFD-fed mice (58). By contrast, in high-fat, high-fructose, high-cholesterol diet-fed mouse models that mimic advanced disease, single-cell RNA sequencing and *in situ* hybridization reveal marked upregulation of *Igfbp5* during the transition from HSCs to myofibroblasts, reflecting its involvement in fibrogenic remodeling during steatohepatitis progression (59). Mechanistically, IGFBP5 promotes survival of activated HSCs by suppressing apoptosis, while simultaneously upregulating profibrotic genes such as collagen Iα1, TIMP1, and MMP1, thereby linking its overexpression to ECM deposition (60).

IGFBP5 also exhibits stage-dependent activity in MASLD. For example, expression in FFA-loaded hepatocytes and HFD-fed mice falls during early steatosis, whereas hepatic overexpression restores metabolic homeostasis by reducing the levels of lipogenic proteins, including SREBP1c, FASN, and ACC; upregulating fatty acid oxidation genes, including *Pparα, Cpt1α*, and *Acox1*; and activating IRS1/AKT and AMPK signaling. These coordinated effects reduce hepatic lipid accumulation and ameliorate liver injury in HFD mice (58). By contrast, in a cellular model that mimics disease progression, *IGFBP5* expression is upregulated during HSC activation and promotes activated HSC survival and profibrotic gene expression in LX-2 human HSCs through IGF-independent mechanisms (60). Hepatic knockdown of *Igfbp5* in myofibroblasts dampens fibrosis in both carbon tetrachloride (CCl_4_) and BDL-induced mouse models by blunting HSC-to-myofibroblast transition. Mechanistically, this occurs through stabilization of the BAT3-TGFβ receptor complex to sustain TGF-β signaling (61); however, a chronic cholangiopathy model shows that overexpression of adeno-associated virus (AAV)-IGFBP5 in hepatocytes reduces hepatic fibrosis, extracellular matrix deposition, inflammation, oxidative stress, and hepatocyte proliferation (62). This suggests a protective effect in this context. In conclusion, available evidence suggests that IGFBP5 may exert stage-dependent effects, with metabolically protective actions reported in early steatotic models and profibrotic effects observed mainly in activated HSCs and fibrotic liver models (58, 60–62).

Additionally, in adults with biopsy-proven MASLD, serum IGFBP5 levels correlate with histological steatosis grade, fibrosis stage, and definite MASH, and can help distinguish advanced fibrosis and definite MASH from other MASLD categories, supporting its use as a noninvasive marker of severity (63).

In summary, IGFBP3 and IGFBP5 are stage-dependent drivers of the intermediate and late stages of MASLD, exerting diverse and often IGF-independent actions. IGFBP3 contributes to fibrogenesis through nuclear signaling and regulation of inflammation, with its serum ratio to IGFI serving as a potential fibrosis biomarker. IGFBP5 actively promotes ECM deposition and HSC survival, linking it directly to the fibrotic process.

## 5 Pathogenic modulators: IGFBP7

While IGFBP3 and IGFBP5 exert context-dependent effects in MASLD, IGFBP7 is a pathogenic modulator that drives insulin resistance, hepatocellular injury, and fibrogenesis.

### 5.1 IGFBP7

IGFBP7, also known as IGFBP-rP1 or mac25, is a secreted ECM-associated glycoprotein. Unlike canonical IGFBPs, IGFBP7 binds to IGFI and IGFII with relatively low affinity, but binds to insulin with high affinity and can inhibit the earliest stages of insulin receptor signaling (65). It also binds directly to the extracellular domain of insulin-like growth factor 1 receptor (IGF1R) to prevent receptor activation by IGF ligands, thereby dampening PI3K-AKT signaling (66). These receptor-proximal actions are complemented by interactions with the ECM, including binding to type IV collagen and the basement membrane via its heparin sulfate domain, which can exert IGF-independent effects on adhesion and signaling (67). Together, these unique features underlie the pathogenic roles of IGFBP7 in regulating proliferation, apoptosis, migration, and tissue remodeling (65–68).

In patients with MASLD, IGFBP7 expression rises with histological disease severity. A biopsy-confirmed study showed that hepatic *IGFBP7* mRNA levels correlate positively with steatosis grade and the NAFLD Activity Score (NAS), which reflects the combined severity of steatosis, lobular inflammation, and hepatocellular ballooning. Higher hepatic expression of *IGFBP7* mRNA is associated with adverse glycemic indices (64). At the intermediate stage of MASLD, IGFBP7 levels rise substantially, with human data showing high expression in serum samples, which effectively distinguishes these stages and shows strong diagnostic performance in cohorts with biopsy-confirmed fibrosis (69, 121). This trend is particularly evident in advanced fibrosis or cirrhosis (F3–F4), where clinical research reveals increased expression of hepatic and circulating IGFBP7. Across multicenter cohorts, higher IGFBP7 levels are closely associated with MASH and advanced fibrosis (69, 122). Consistent with this, preclinical findings demonstrate that upregulation of IGFBP7 is linked to hepatic injury processes, enabling enhanced prediction of new fibrosis onset (123). In addition, animal studies have shown increased levels of *Igfbp7* mRNA. In HFD-fed mice, both hepatic and circulating IGFBP7 increase, while in zebrafish models of MASLD, expression of *igfbp7* is also upregulated during steatohepatitis and fibrosis (29, 70, 71).

IGFBP7 contributes to MASLD progression by binding to insulin with high affinity, thereby impairing insulin receptor signaling. It also promotes ferroptosis and HSC activation. In HFD-fed mice, AAV-shRNA-mediated silencing of *Igfbp7* reduces hepatic triglyceride content and restores insulin signaling by enhancing phosphorylation of IRS1, AKT, and glycogen synthase kinase 3 beta (GSK3β), thereby ameliorating insulin resistance and normalizing downstream metabolic control (71). In the zebrafish model of MASLD, *igfbp7* depletion suppresses nuclear receptor coactivator 4 (NCOA4)-mediated ferritinophagy and ferroptosis, thereby mitigating lipid peroxidation, inflammation, and fibrosis (70). Moreover, in cell and rodent models, increased expression of IGFBP7 protein by activated HSCs promotes further HSC activation and hepatocyte apoptosis through TGF-β-SMAD2/3 signaling, thereby enhancing fibrotic remodeling (72, 73). These mechanisms align with clinical observations that circulating levels of IGFBP7 are higher in individuals with T2DM, a major risk factor for MASLD (124). In conclusion, IGFBP7 impairs insulin/IGF signaling and fosters ferroptotic and fibrotic programs, thereby promoting disease progression from steatosis to MASH and advanced fibrosis.

Notably, IGFBP7 can serve as a biomarker for late-stage MASLD. In blood, translational proteomics identified IGFBP7, together with scavenger receptor cysteine-rich family member with 5 domains and semaphorin 4D, as a biopsy-conferred biomarker panel that accurately differentiates the fibrosis stages of MASLD. Meanwhile, because circulating IGFBP7 is lower at the early fibrosis stage (F0–F1) than at the advanced stage of the disease, this three-analyte panel contributes meaningfully to non-invasive classification when combined with complementary proteins (69). Additionally, a recent proteo-transcriptomic study has shown that plasma IGFBP7 is a robust biomarker of advanced fibrosis and an independent predictor of liver-related outcomes, outperforming Fibrosis-4 and AST to platelet ratio index and improving risk stratification when combined with the Chronic Liver Disease_lab_ score (125).

In summary, IGFBP7 promotes MASLD by sequestering insulin, impairing hepatocellular insulin signaling, promoting ferroptosis, and driving HSC activation through the TGF-β-SMAD2/3 pathways. Both human biopsy data and animal studies confirm the contribution of IGFBP7 to lipid peroxidation, inflammation, and ECM deposition. By amplifying metabolic dysfunction and fibrotic responses, IGFBP7 represents a pathogenic modulator of MASLD pathogenesis.

## 6 Potential regulators: IGFBP4 and IGFBP6

Among all IGFBPs, IGFBP4 and IGFBP6 have received relatively limited attention in the context of MASLD research; however, emerging findings suggest that these proteins may play context-specific roles in linking systemic metabolic stress, innate immunity, and hepatocellular defense responses to MASLD. Based on current evidence, IGFBP4 and IGFBP6 may regulate inflammation and stress adaptation at the early or intermediate disease stages.

### 6.1 IGFBP4

IGFBP4 is a 28 kDa secreted protein that binds to IGFI and IGFII with high affinity, primarily modulating ligand availability to IGF1R (30). Structurally, the human *IGFBP4* gene contains a TATA box-driven cAMP-responsive promoter, with the transcription start sites located downstream of the TATA box (30). This promoter architecture is unique among IGFBPs, which generally lack such TATA-dependent cAMP-regulated transcriptional control. In addition, *IGFBP4* undergoes IGF-dependent proteolytic cleavage by pregnancy-associated plasma protein-A (PAPP-A), a post-translational regulatory process unique within the IGFBP family (31). These different structural and regulatory specificities allow IGFBP4 to regulate diverse biological processes, such as inhibition of Wnt-β-catenin signaling, to promote cardiomyocyte differentiation of stem cells (126, 127), suppression of neural progenitor cell proliferation through IGF1R pathways (128), and impairment of osteogenic differentiation of mesenchymal stem cells via the ERK and SMAD pathways (129).

Expression of IGFBP4 demonstrates tissue-specific roles. Circulating levels increase under hypoxic conditions, as evidenced by elevated serum IGFBP4 in cases of OSA (130). In primary rat hepatocytes, IL-6 induces hepatic IGFBP4 mRNA and protein expression, an effect attenuated by TNF-α and IL-1β. *In vivo*, administration of IL-6 to rats increases liver *Igfbp4* mRNA levels by about 3-fold, which is mirrored by elevated serum IGFBP4 levels (131). In addition, MYB-binding protein 1A mediated epigenetic silencing of IGFBP4 through hypermethylation of promoter CpG islands has been reported in HCC (132). These observations suggest that IGFBP4 may respond to hypoxic, inflammatory, or epigenetic cues in liver-related contexts, but whether such regulation occurs in MASLD remains unknown.

Overall, current knowledge of IGFBP4 in MASLD remains highly limited. Most available evidence is derived from non-MASLD systems, including hypoxia, inflammatory stimulation, and cancer models. Therefore, the role of IGFBP4 in MASLD requires validation in human cohorts and experimental models.

### 6.2 IGFBP6

IGFBP6, a classical IGF-binding glycoprotein with markedly higher affinity for IGFII than for IGFI, exhibits roles in immune regulation (32, 74). Structurally, IGFBP6 differs from other IGFBPs due to its unique genomic organization. The human *IGFBP6* gene, located on chromosome 12q13, spans 4.7 kb; it comprises four exons and lacks TATA or CAAT box elements in its promoter region, which may potentially influence its transcriptional regulation compared with that of other IGFBPs with canonical promoter sequences (32). A study in a biopsy-based human MASLD cohort showed that despite fibrosis stage showing a stronger association with IGFBP7, hepatic *IGFBP6* mRNA levels increased along with steatosis severity and NAS (64).

Findings from liver fibrosis models suggest that IGFBP6 may participate in fibrogenic responses. For example, in experimental models using LX-2 human HSCs and mouse models of liver fibrosis, *IGFBP6* is upregulated in HSCs and promotes fibrogenesis through the TGF-β/SMAD pathway, thereby contributing to ECM deposition (75). Further research is essential to delineate the effects of IGFBP6 on hepatic lipid metabolism, insulin sensitivity, and its potential as a biomarker or therapeutic target for MASLD. Major research gaps include the lack of studies examining IGFBP6 in MASLD models, its direct effects on hepatocyte lipid accumulation, and human data correlating IGFBP6 levels with MASLD severity or metabolic risk factors.

In conclusion, IGFBP4 and IGFBP6 remain under-explored in the context of MASLD, with existing insights derived from fibrosis, vascular, and cancer models rather than liver-specific studies. Although these findings raise the possibility that IGFBP4 and IGFBP6 may be relevant to metabolic stress, inflammation, or fibrogenic responses, direct evidence in MASLD remains limited. Their roles should therefore be regarded as hypothetical and require validation in MASLD-specific human and experimental studies.

## 7 Conclusion

IGFBPs are increasingly recognized as active regulators of MASLD, with functions extending beyond their canonical roles as IGF-binding proteins. IGFBP1 and IGFBP2 appear to exert metabolically protective effects during early disease, whereas IGFBP3 and IGFBP5 may have dual roles, with protective actions in steatotic contexts but potential profibrotic effects during disease progression. IGFBP7 shows a predominantly pathogenic profile by impairing insulin signaling, promoting ferroptosis, and facilitating hepatic stellate cell activation. In contrast, the roles of IGFBP4 and IGFBP6 remain insufficiently defined and require further validation in MASLD-specific models.

Thus, the effects of IGFBPs are highly stage-dependent and context-specific, shaping the transition from hepatic steatosis to steatohepatitis, fibrosis, and cirrhosis. Because MASLD involves complex metabolic, inflammatory, and fibrogenic networks, IGFBPs likely influence disease progression through multiple interconnected signaling pathways. A consolidated summary of MASLD-associated IGFBP pathways is presented in **Table 3**, providing a framework for future mechanistic and therapeutic investigations.

**Table 3:** Representative signaling pathways regulated by IGFBP1–7 in MASLD progression.

IGFBP	Key pathway	Regulations	Roles		Reference
IGFBP1	PPARα/CPT1α/PGC1α axis^b^	Upregulated	↑ β-oxidation	Protective	(35)
	SREBP1/FASN/ACC axis^b^	Downregulated	↓ Lipogenesis		(35)
	ERK signaling^b^		↓ Inflammation		(35)
	NF-κB signaling^b^				(35)
IGFBP2	IRS1/CPT1α signaling	Upregulated	↑ β-oxidation	Protective	(114)
	EGFR-STAT3 signaling^b^	Downregulated	↓ Lipogenesis		(43)
IGFBP3	JNK signaling	Downregulated	↓ Stress/inflammation	Protective	(51)
	NF-κB signaling				(51)
	Pro-fibrotic signaling in HSCs^b^	Upregulated	↑ Fibrogenesis	Pathogenic	(57)
IGFBP5	PPARα/CPT1α/ACOX1 axis	Upregulated	↑ β-oxidation	Protective	(58)
	AMPK/IRS1/AKT pathway	Upregulated	↓ Lipogenesis ↑ Insulin sensitivity		(58)
	SREBP-1c/FASN/ACC axis	Downregulated	↓ Lipogenesis		(58)
	TGF-β/SMAD signaling (HSCs)^b^	Upregulated	↑ Fibrogenesis	Pathogenic	(60, 61)
IGFBP7	TGF-β/Smad2/3 signaling^b^	Upregulated	↑ Fibrogenesis	Pathogenic	(73)
	NCOA4 pathway^b^	Upregulated	↑ Ferroptosis		(70)
	IGF1R/PI3K-AKT pathway^a^	Downregulated	↑ Insulin Resistance		(66)
	Insulin receptor signaling^b^	Downregulated			(71)
IGFBP6	TGF-β/SMADs pathway	Upregulated	↑ Fibrogenesis	Pathogenic	(75)
IGFBP4	No direct MASLD-specific pathway established				

This table summarizes the major intracellular signaling pathways modulated by individual IGFBPs, and their functional consequences at different stages of MASLD. Superscript a indicates IGF-dependent signaling, whereas superscript b indicates IGF-independent signaling. Pathways without a superscript annotation have unclear IGF dependency. IGFBP1 and IGFBP2 primarily act as protective modulators by enhancing β-oxidation, insulin sensitivity, and suppressing lipogenesis and inflammation. IGFBP3 and IGFBP5 exhibit dual and stage-dependent roles: their early-stage downregulation of stress and lipogenic pathways is hepatoprotective, whereas late-stage activation of integrin and TGF-β-driven signaling promotes fibrogenesis. IGFBP7 is a pathogenic modulator that enhances fibrogenesis, ferroptosis, and insulin resistance. IGFBP6 mainly engages the TGF-β/SMAD axis, contributing to fibrogenic and pathogenic outcomes, while IGFBP4 currently lacks defined signaling associations in the context of MASLD. Upward arrow ↑ indicates an increase or upregulation, whereas downward arrow ↓ indicates a decrease or downregulation in pathway activity or biological processes shown in the Roles column.

Together, the distinct yet overlapping roles of IGFBPs in insulin sensitivity, lipid metabolism, immune responses, and fibrogenesis highlight their potential as diagnostic biomarkers and therapeutic targets. Future studies should clarify the temporal, spatial, and cell-specific dynamics of each IGFBP during MASLD progression, while longitudinal human studies will be essential to validate these dynamic patterns, define clinically relevant mechanisms, and evaluate the integration of IGFBP-based markers into existing risk models. Translational strategies, such as IGFBP2 analogs or IGFBP7 inhibition, may ultimately support precision therapies targeting both the metabolic and fibrotic axes of MASLD.
